# The high dose of vitamin D supplementation combined with yoga training improve the leukocytes cell survival-related gene expression in breast cancer survivors

**DOI:** 10.1186/s12986-021-00607-7

**Published:** 2021-08-28

**Authors:** Vahid Khedmati Zare, Maedeh Javadi, Sadegh Amani-shalamzari, Mojtaba Kaviani

**Affiliations:** 1 Department of Exercise Physiology, Faculty of Physical Education and Sports Science, Kharazmi University, Tehran, Iran; 2 School of Nutrition and Dietetics, Faculty of Pure and Applied Science, Acadia University, Wolfville, NS Canada

**Keywords:** Physical activity, Immune system, Flexibility, NF-κB, p53, Bcl2/Bax ratio

## Abstract

**Background:**

This study aimed to examine the effect of yoga training combined with vitamin D supplementation on the expression of survival-related genes in leukocytes and psycho-physical status in breast cancer survivors.

**Methods:**

Thirty breast cancer survivor women (age, 48 ± 8 yrs) were randomly assigned into three groups: high dose (4000 IU) of vitamin D supplementation (HD) (n = 10); yoga training with a high dose of vitamin D (Y + HD); (n  = 10); yoga training with a low dose (2000 IU) of vitamin D (Y + LD) (n = 10). Participants performed the Hatha yoga style twice a week. Blood samples and a battery of psychological and physical tests were taken before and after the completion of interventions. Expression of p53, NF-κB, Bcl2, and Bax genes was measured in leukocytes.

**Results:**

Body fat percentage (ηp2 = 0.36), shoulder flexibility (ηp2 = 0.38), Rockport walk tests (ηp2 = 0.49), and anxiety (ηp2 = 0.52) were significantly improved in both the Y + HD and Y + LD groups compared to the HD group (*p* < 0.05). P53 was significantly over-expressed in the Y + HD group while Bcl2 upregulated in both the Y + HD and Y + LD groups. NF-κB and Bax expression downregulated in all groups but were not statistically significant.

**Conclusion:**

yoga training combined with low and high doses of VD improved physical fitness and psychological measures while only in combination with a high dose of VD positively modified the leukocytes cell survival-related gene expression.

## Introduction

Breast cancer (BC) treatments such as chemotherapy and radiotherapy typically cause side effects on psychological, physical, and physiological health in patients. Research has shown that BC survivors are at higher risk of developing psychiatric disorders, especially depression, stress, and anxiety [1], as well as impairment of the physical performance, such as strength and aerobic fitness [2]. In addition, some of the physiological functions of BC survivors, such as immune responses, are impaired by cancer treatments [3, 4]. Immunosuppression is one of the significant side effects of BC treatments [5]. A study reported that recovery of numerous immune parameters following BC therapies is delayed; hence, attenuated immune systems are involved in the recurrence of the disease and prolong cancer recovery [5].

Immune cell survival is a highly sophisticated process regulated by the balance between apoptotic and anti-apoptotic genes. P53 is an anti-apoptotic gene that promotes cell survival by inducing reversible cell cycle arrest [6]. P53 regulates immune cells' function; however, p53 activity in leukocytes of BC survivors has not been studied. It was reported that the loss of p53 in the immune cells of rodents leads to increased susceptibility to tumor growth [7]. On the other hand, NF-κB is a crucial regulator of several biological processes such as immunity, inflammation, and apoptosis, which could upregulate various pro-inflammatory and cell death genes [8]. NF-κB translates stress into inflammation and is a link between inflammation and cancer [9]. It is involved in the cell apoptosis process via suppressing the anti-apoptotic gene, Bcl2, and upregulating apoptotic gene, Bax; hence NF-κB decreases the Bcl2/Bax ratio [10, 11]. It was also reported that chemotherapy was associated with NF-κB activation in BC tissue and leukocytes [12], and enhanced NF-κB activity was paralleled with physical fatigue [12]. In contrast, P53 counteracts the effects of NF-κB [13] and increases cell survival by inducing reversible cell cycle arrest [6]. Therefore, the balance between NF-κB and p53 is involved in cell survival.

Complementary therapies, including nutrient supplements and exercise training, involve controlling gene expression and are a new approach to managing breast cancer symptoms. Cancer patients have been shown to be malnourished, which can lead to worsening their condition [14]. Thus, antioxidants [15] and vitamins [16] supplements are recommended for cancer patients. These supplements contain nutritious and polyphenolic compounds that could regulate genes' expression involved in inducing and progressing chronic diseases. Research has shown vitamin D (VD) deficiency is a common condition in women with the later-stage disease and those who received radiation therapy [17], which is associated with increased BC mortality rates [17, 18]. Thus, most BC survivors receive different doses of VD. Vitamin D plays some physiological functions, such as cell cycle regulation, by affecting the p53 gene expression [19] and inhibits the activation pathway of NF-κB in human leukocytes [20, 21]. Besides, VD plays a role in the cell survival cycle by regulating the Bcl2/Bax ratio [22]. Research has shown that VD exerts a growth inhibitory effect on breast cancer cells, and the treatment of breast cancer cells with VD led to the upregulation of 51 genes, 40% of which were involved in the cell cycle and apoptosis [23]. It has been shown that a high dose of VD supplementation is safe and has favorable effects on insulin-like growth factor signaling with no significant change in mammographic density in women at higher risk of BC [24].

A non-pharmaceutical approach to mitigate the side effects of cancer and its treatments is physical activity. In systematic reviews, improvements in physical fitness and psychological health of cancer survivors with yoga were reported [25, 26]. Yoga is a physical, mental, and spiritual exercise that integrates mind, body, and spirit to improve psychological and physical health by reducing stress [27]. Yoga exercises include physical exercises (asanas), breathing techniques (pranayama), and meditation (Dyana). Yoga has recently been adapted as a complementary treatment to reduce chronic disease-induced stress [28], especially for BC-related mental health complications. BC-related chronic stress has been shown to induce chronic inflammation, leading to the suppression of the immune system. Mechanistically, T lymphocytes were suppressed through a high cortisol level [29], potentially delaying the treatment process. Knowingly, yoga therapies could reduce inflammatory responses [30] and improve various immunological parameters of cellular immunity [31]. Still, the molecular mechanisms of these benefits are poorly understood. It was demonstrated that yoga intervention might downregulate NF-κB in leukocytes of dementia patients [32], yet, the molecular mechanisms and yoga practice on the immune cells in BC survivors are unclear.

Recent studies have suggested the possible involvement of exercise training in reducing the side effects of high doses of antioxidant supplementation in breast cancer tumors [33]. However, considering the immune system's responses, the impact of simultaneous exercise training and a high dose of VD intake on leukocyte survival in BC survivors have not been elucidated. Overall, given that a high dose of vitamin D is safe for women at higher risk of BC [24] and recommended for BC survivors to reduce inflammatory markers; yoga also has a substantial effect on this process; we hypothesized that combining yoga exercise training and high VD dose could modulate the gene expression involved in the survival of immune cells and improve the immune system of BC survivors. Moreover, we examined whether the combination of yoga training and low VD dose could have the same beneficial effects. At last, we assessed the effects of these interventions on anthropometric, physical, and psychological features.

## Materials

### Study design

In a randomized trial with pre and post-test, the effectiveness of a 12-week yoga training with VD supplementation was examined. Eligible BC survivors were recruited to take part in the study under the supervision of their physicians. A week before the intervention, VD levels and pre-tests were completed, and physicians selected qualified individuals. Then, randomization based on the initial VD levels was conducted by the third person who was not in the research group. Before and after the intervention, blood sampling, anxiety questionnaire, and physical fitness tests including, Rockport walk and handgrip strength tests, were measured. Gene expression of P53 and NF-κB were measured in blood samples.

### Participants

Breast cancer survivors referred to Shohadaye Tajrish hospital (Tehran, Iran) were contacted for this study. Survivors who had completed chemotherapy and radiotherapy five years ago and were exposed to hormone therapy, and did not have any acute medical conditions (cardiovascular diseases, diabetes) were eligible to participate. Also, individuals were selected who did not have any experience of yoga training and had a high-dose VD supplementation, and were physically able to perform yoga exercises. Thirty-three BC survivor women who met the inclusion criteria volunteered to participate in the survey, but we only analyzed the data of 30 participants (age: 47.90 ± 7.95 years; height: 160.93 ± 6.12 cm, body mass: 72.62 ± 11.72 kg). One participant was excluded from the study due to a change in a medical situation; one did not participate in more than four consecutive training sessions, one failed to complete the post-test. Participants were randomly assigned into a high dose (4000 IU) of VD supplementation (HD) group (n = 10), yoga with a high dose (4000 IU) of VD (Y + HD) group (n = 10), and yoga with a low dose (2000 IU) of VD (Y + LD) group (n = 10). Due to ethical reasons, it was impossible to have a group that only practices yoga because BC survivors were prescribed to consume VD. After being informed of the benefits and risks of research, written consent was obtained from the participants. A sample size calculation was performed using G*Power Software version 3.1.9.6 (Düsseldorf, Germany) to determine the number of participants in each group. The estimated number of patients needed to assume a rejection criterion of 0.05 and 0.85 (1-beta) power and a large effect (f = 0.65) was ten persons per group, depending on the statistical test used. The Ethics committees approved all research procedures for the Sport Sciences Research Institute of Iran (approval number: IR.SSRI.REC.1398.111) were conducted under the Declaration of Helsinki.

### Vitamin D supplementation

Participants in the HD and YHD groups received VD tablets containing 4000 IU daily, and those in the YLD group consumed 2000 IU daily. They have received a pack of 10 tablets every 10 days to take every day at a given time by receiving an alarm from the WhatsApp group.

### Yoga protocol

Participants in the yoga groups performed yoga practice twice a week for 12 weeks. The duration of the yoga program started with 60-min and progressively increased to 90 min over the course. One certified yoga teacher conducted yoga sessions. Yoga exercises were selected from the Hatha yoga style and included asana (physical postures), pranayama (breath control), and Dyana (meditation). The yoga protocol is presented in Table 1. After each yoga session, the participants were asked to express their effort and exertion (the Borg rating of perceived exertion (RPE) scale (6–20 score)) of the yoga session [34]. As the training progressed, more complex and more challenging movements were selected, as well as the movements were repeated more to increase the load of workouts.

**Table 1:** Yoga protocol

Pranayama	Breathing and moving (Yoga Mudra)
Asana	Child's Pose (Balasana)
Asana (Marjaryasana cycle)	Cradle the leg Pose (Hindolasana), Cradle Pose knees to chest (Hindolasana), bridge pose (Setu Bandha), Cobra Pose (Bhujangasana), Child's Pose (Balasana), Cradle Pose knees to chest (Hindolasana), Cow Pose (Bitilasana), Cat Pose (Marjaryasana), Bird Dog Pose (Parsva Balasana), Gate Pose (Parighasana), Side Plank Pose Variations on knee (Vasisthasana), Cow Pose (Bitilasana), Cat Pose (Marjaryasana), Hero Pose (Virasana), Staff Pose (Dandasana), Corpse Pose (Savasana)
Pranayama	Respiratory coordination
Asana	Like burpee (Surya namaskar or sun salutation)
Asana	Bound Angle Pose (Baddha Konasana)
Asana	Mill Churning Pose (Chakki Chalanasana)
Asana	Chair Pose (Utkatasana)
Asana	Reclining Bound Angle Pose (Supta Baddha Konasana)
Asana	Cobra Pose (Bhujangasana)
Asana (kriya cycle)	Triangle Pose (Utthita Trikonasana), Waist Rotating Pose Variation A Yoga (Katichakrasana), Big Toe Pose (Padangusthasana), Mill Churning Pose (Chakki Chalanasana), Staff Pose with front lateral raises (Dandasana), Revolved Hero Pose Yoga (Parivrtta Virasana), Cobra Pose (Bhujangasana), Boat Pose (Paripurna Navasana)
Asana	Locust Pose (Salabhasana)
Asana	Half Wind Relieving Pose (Ardha Pavana Muktasana)
Asana	Wind-Relieving Pose (Pavanamuktasana)
Asana	Twisting Posture ( suptaVakra Asana)
Dyana (meditation)	Corpse Pose (Savasana)

## Measurements

### Physical measurements

Anthropometric measures, including height and body mass, were measured using a height scale (Seca 206, Hamburg, Germany) and a digital body weight scale (Seca 803, Hamburg, Germany), respectively. Moreover, body fat percentage (BF %) was estimated by measurement of subcutaneous fat of seven skinfold sites based on Jackson and Pollock's instructions [35] using a Lange skinfold caliper (beta Technology Inc., Cambridge, MD USA).

### Rockport walk test (RWT)

The RWT test, a one-mile self-paced walking test, was used to measure aerobic fitness [36]. The test measures the time lasted to walk one mile as quickly as safely. Four cones were placed in the corners of the futsal court, and every meter was marked between the cones. Participants walked at a rate suitable to their fitness for one mile while wearing a polar heart rate monitor Polar RC3, Polar Electro Oy, Kempele, Finland), and they were allowed to stop or change their paces.

### Shoulder range of motion

The range of active shoulder abduction in the side of surgery was measured by a Leighton flexometer (Spokane, WA, USA) [37]. Participants stood near a pillar, and their head, trunk, and hip were fixed with straps. A flexometer was attached to their arm; the dial at 0 degrees was considered when the hand hanged on the side of the body. Then, participants elevated their arms in the strict coronal plane as far as they could. At the end-range point, the measurement was documented. The best of the three trials was considered.

### Anxiety

The Beck Anxiety Inventory (BAI) was used to assess anxiety [38]. The questionnaire consists of 21 items that evaluate the clinical symptoms of anxiety in the past month. All questions had a similar weight and score on a scale value of 0 (not at all) to 3 (severely). A higher score indicates a more severe anxiety level.

### Cortisol

Blood samples were collected from the antecubital venous after 10 to 12 h of fasting between 7 and 8 am before and after the interventions. Blood samples were centrifuged at 3000 rpm for 15 min, and serum was separated from the blood samples using a suspension technic and stored at a − 20 °C freezer until further biochemical analysis. Serum cortisol was measured using an enzyme-linked immunosorbent assay (ELISA) kit (Monobind, Inc. Lake Forest, CA, USA).

### Gene expression

Blood samples in tubes containing anticoagulants (EDTA) were centrifuged at 3000 rpm in a 4 °C centrifuge for 10 min. The buffy coat layer was removed from the blood samples using a suspension technic and stored at a – 70 °C freezer. RNA samples were isolated from buffy-coats (white blood cells–enriched blood samples) extracted using the total RNA extraction Kit (Takara, Japan). The total RNA concentration was assessed using a NanoDrop ND-1000 (NanoDrop Technologies/Thermo Scientific, Wilmington, DE, USA), and cDNA synthesis was performed using the Takara cDNA synthesis kit (Takara, Japan) according to the manufacturer's instructions. Real-time PCR was performed using the SYBR Green Master Mix kit (Ampliqon, Denmark). The thermal cycling program was as follows: 94 °C for 5 min followed by 40 cycles of 95 °C for 30 s, 54 °C for 45 s, and 72 °C for 30 s. We used GAPDH mRNA for the normalization of the gene expression analysis. The sequence of PCR primers used for the amplification of the protein-coding genes were as follow: p53 forward “TGACTGTACCACCATCCACTA”, p53 reverse “AAACACGCACCTCAAAGC”; NF-κB forward “CTTGGGTGCTGATGTCAATG”, NF-κB reverse “GGAGAATAGCCCTGGTAGG”; BAX forward “TGACTGTACCACCATCCACTA” BAX reverse “AAACACGCACCTCAAAGC”; Bcl2 forward “CCCTGTGGATGACTGAGTAC” Bcl2 reverse “GAGAAATCAAACAGAGGCCG”; GAPDH forward “CCCTTCATTGACCTCAACTACATG” GAPDH reverse “TGGGATTTCCATTGATGACAAGC”. The fold change expression was calculated using the REST-2009 software.

### Statistical analysis

The Statistical Package of Social Sciences (SPSS, IBM, v19) was used to analyze the data; parametric data are expressed as mean ± standard deviation (SD), and non-parametric data are expressed as median (25th-75th interquartile range). Analysis of covariance (ANCOVA) was used to explore the main effects of different interventions on the variables. Pre-test data was used for the covariate. If significant effects were found, Bonferroni post-hoc tests were performed. In addition, data of gene expression changes were calculated by 2^−∆∆CT^ and analyzed by ANOVA. To define the magnitude and direction of the linear relationship between survival-related gene expression with cortisol, anxiety, and VD, the bivariate Pearson correlation coefficient (r) was calculated on the magnitude of changes. The magnitude of changes was determined by subtracting post-test values from pre-test values. Effect sizes (ES) were also computed as the change score divided by the SD of the change score to examine the magnitude of differences while controlling for the influence of the sample size [39] with < 0.2 considered as a small ES, 0.2–0.5 as a moderate ES, 0.5–0.8 as a large ES, and > 0.8 as a very large ES. The percentage changes were calculated by subtracting the pre-test from the post-test divided by the pre-test multiplied by100. The significance level was set at *p* ≤ 0.05.

## Results

Table 2 provides descriptive data of performance and psychological indicators as well as the changes in VD levels. There were no significant differences between groups in all variable levels at the baseline (*p* > 0.05). A 12-week VD supplementation period led to a significant increase in VD levels, and a significant difference was observed between both groups who received high VD dose and Y + LD group (F = 6.5, *p* = 0.005, ηp2 = 0.33). A 12-week yoga training significantly decreased body fat percentage, as evidenced in the Y + HD and Y + LD groups compared to the HD group with moderate effect size (F = 7.2, *p* < 0.003, ηp2 = 0.36). We observed that there were significant differences between groups at shoulder flexibility (Ipsilateral: F = 7.8, *p* = 0.002, ηp2 = 0.38; Contralateral: F = 6.4 *p* = 0.005, ηp2 = 0.33), and Rockport walking test with moderate effect size (F = 12.4, *p* < 0.001, ηp2 = 0.49). The Bonferroni post hoc test showed that shoulder flexibility and Rockport walk tests significantly improved both the Y + HD and Y + LD groups than the HD group (*p* < 0.05).

**Table 2:** Performance and psychological indicators of participants before and after performing the intervention

Variable	Group	Pre	Post	% change	*P* within	*P* between
Vitamin D (IU)	HD	41.2 ± 16.2	53.5 ± 15.9	34.62	0.001	0.005
	Y + HD	44.8 ± 13.1	57.5 ± 12.3	32.17#	0.001	
	Y + LD	43.4 ± 15.1	49.3 ± 16.2	15.69*	0.001	
Body fat percentage (%)	HD	37.0 ± 4.4	36.8 ± 4.3	− 0.52	0.343	0.003
	Y + HD	34.8 ± 3.3	33.4 ± 2.9	− 3.94*	0.003	
	Y + LD	37.0 ± 4.1	35.3 ± 4.3	− 4.67*	0.001	
Rockport walk test (s)	HD	1129.7 ± 118.0	1132.2 ± 106.8	0.33	0.774	0.001
	Y + HD	1106.1 ± 84.5	1048.9 ± .32.2	− 5.00*	0.018	
	Y + LD	1112.2 ± 46.9	1059.1 ± 46.9	− 4.75*	0.001	
*Shoulder flexibility (˚)*						
Ips	HD	125.4 ± 18.0	125.4 ± 17.8	0.04	0.991	Ips:
Con		231.1 ± 19.1	231.1 ± 18.9	0.01	0.991	0.002
Ips	Y + HD	125.9 ± 14.0	139.8 ± 12.8	11.9*	0.013	Con:
Con		234.1 ± 14.0	220.2 ± 12.8	− 5.8*	0.013	0.005
Ips	Y + LD	127.7 ± 11.7	138.8 ± 8.9	9.1*	0.001	
Con		230.8 ± 10.8	220.7 ± 8.5	− 4.3*	0.001	
Anxiety (U.A)	HD	16.3 ± 9.3	16.1 ± 9.1	0.02	0.343	0.001
	Y + HD	17.3 ± 9.5	8.1 ± 6.4	− 56.53*	0.003	
	Y + LD	14.2 ± 9.1	6.8 ± 5.1	− 49.44*	0.002	
Cortisol (μg/dl)	HD	16.2 ± 5.8	21.9 ± 9.4	33.5	0.073	0.397
	Y + HD	16.3 ± 4.7	19.8 ± 6.5	19.3	0.084	
	Y + LD	20.0 ± 6.5	22.2 ± 5.3	15.3	0.198	

HD, high dose of vitamin D group; Y + HD, yoga with a high dose of vitamin D group; Y + LD, yoga with a low dose of vitamin D group. Ips, Ipsilateral, Con; Contralateral

*Significant difference with HD group

^#^Significant difference with Y + LVD group

A significant difference in anxiety was observed between groups with a moderate effect size (F = 14.2, *p* < 0.001, ηp2 = 0.52). A 12-week yoga training was more useful to reduce anxiety in both the Y + HD and Y + LD groups than in the HD group. There were no significant changes in anxiety in the HD group (*p* > 0.05) (Table 1). Further, there was no significant difference between groups at the cortisol level with a relatively low effect size (F = 0.95, *p* = 0.397, ηp2 = 0.07) (Table 2).

**Fig. 1:**
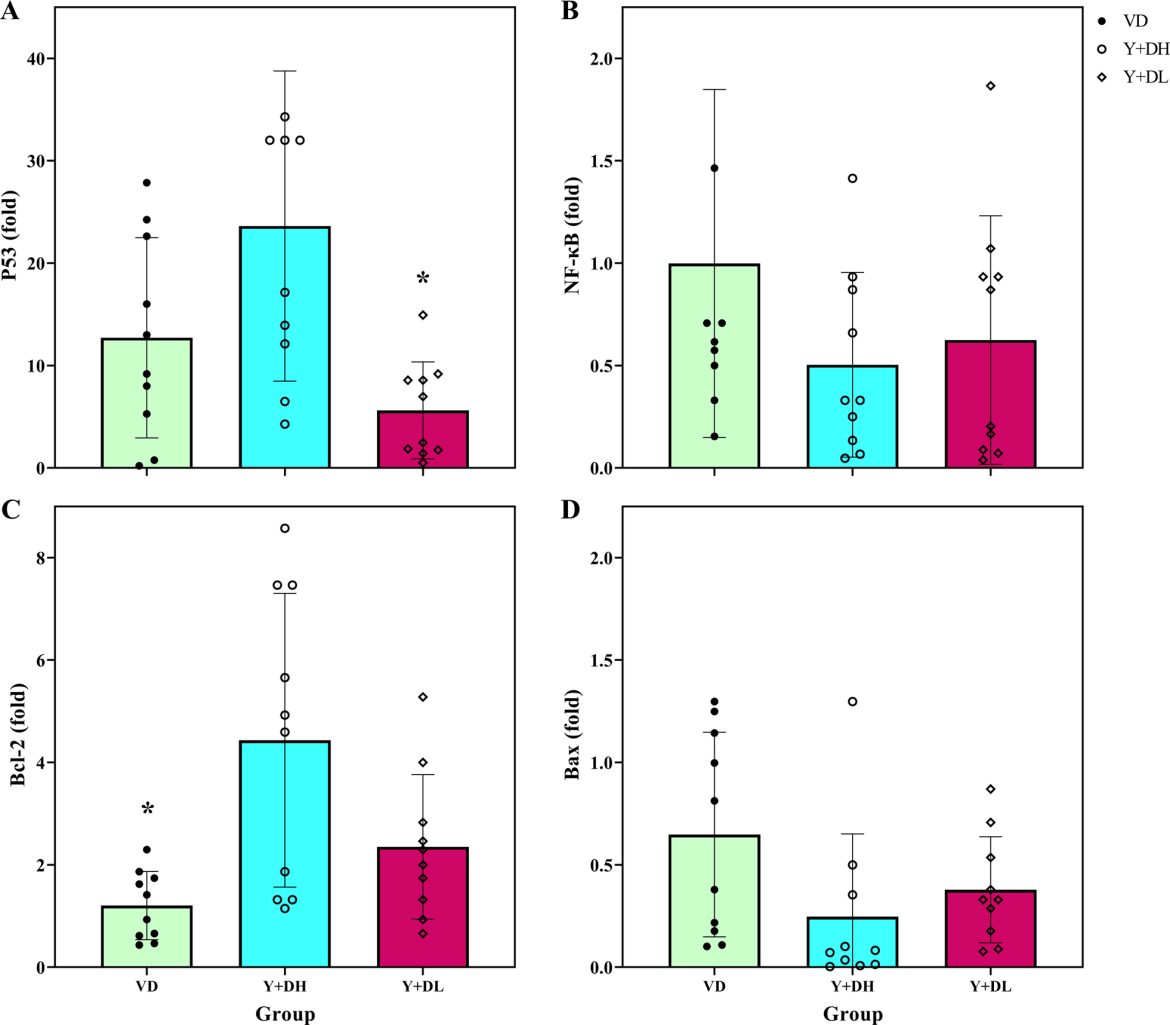
Gene expression of p53 (**a**), Bcl2 (**b**), NF-κB (**c**) and Bax (**d**) at baseline and after intervention in peripheral blood cells. VD: high dose of vitamin D, Y + DL: yoga with a low dose of vitamin D, Y-DH: yoga with a high dose of vitamin D. *Significant difference from pre-to post intervention

Figure 1a shows P53 was over-expressed in all groups after the intervention; a significant difference was observed in leukocyte's P53 expression between the groups (F = 7.1, *p* = 0.003). The Bonferroni post hoc test showed a significant difference was between the Y + HD group and Y + LD (*p* = 0.002).

Data appeared that leukocyte's NF-κB expression was down-regulated in all groups following the intervention, but this change was not statistically significant between groups (F = 1.5. *p* = 0.231) (Fig. 1b).

A 12-week of yoga training led to up-regulate Bcl2 expression in all groups after intervention. There was a significant difference in leukocyte's Bcl2 expression between the groups) F = 7.5, *p* = 0.003). The Bonferroni post hoc test showed a significant difference was between the Y + HD group and HD (*p* = 0.002) (Fig. 1c).

We observed that leukocyte Bax expression was down-regulated in all groups following the intervention, but there was no significant difference between groups (F = 2.6. *p* = 0.091) (Fig. 1d).

Table 3 presented the correlation between changes in leukocyte survival-related gene expressions and anxiety, VD, and cortisol levels after the intervention. Anxiety level had a significant positive relationship with NF-κB and Bax expression and was negatively associated with p53 and Bcl2 expression. Cortisol level had a significant positive correlation with NF-κB expression and a negative correlation with p53 and Bcl2 expression. In addition, VD only correlated with p53 and Bcl2 expression.

**Table 3:** Pearson correlation coefficient (r) between changes in survival-related gene expression and anxiety, vitamin D, and cortisol after the intervention

Variables	∆ anxiety	∆ VD	∆Cortisol
P53	− 0.43*	0.79*	− 0.42*
Bcl2	− 0.64*	0.54*	− 0.54*
NF-κB	0.39*	− 0.29	0.62*
Bax	0.42*	− 0.29	0.15

VD, vitamin D

*Significant correlation (*p* < 0.05)

The intensity of initial sessions was considered low, but a moderate intensity was achieved towards the protocol's end. The range of the Borg scale was 8–14.

## Discussion

The main aim of this research was to evaluate the efficacy of a 12-week yoga training combined with the high dose of VD supplementation on physical performance and genes involved in the survival of immune cells of BC survivors. The high dose of VD supplementation led to significant increases in VD level than the low VD dose. The findings revealed that yoga training combined with the high VD supplementation improved anxiety and the physical fitness of BC survivors, not the high VD amount alone. Participants who received the high dose of VD showed up-regulation of leukocytes P53 & Bcl2 genes involved in cell survival. A down-regulation trend was observed in apoptosis genes (NF-κB & Bax) that were not statistically significant between groups. No change in the circulatory level of cortisol was observed. Moreover, our findings demonstrated that a combination of yoga with a low dose of VD supplementation improved anxiety and the selected physical fitness indicators but did not show synergistic effects on gene expression involved in cell survival in peripheral blood cells. Therefore, our findings indicate that BC survivors could benefit from yoga training combined with a high dose of VD supplementation, as evidenced by improvements in physical and psychological measures and gene expression involved in cell survival in leukocytes.

Cancer-related fatigue has been shown to negatively affect physical activity, leading to adverse changes in body composition. Our finding showed that a 12-week yoga training reduced body weight and body fat percentage compared to the HD group, which is supported by the previous studies showing that regular yoga training can reduce body fat and positively affect body composition [40–42]. Littman et al. [41] reported body fat and waist circumference reduced in overweight and obese BC survivors following a six-month yoga training. Exercise training and healthy nutrition reduce body weight by lowering abdominal and subcutaneous fat via increasing metabolic expenditure, leading to improved body composition [43]. Therefore, yoga seems to be effective in weight loss and reducing body fat percentage.

Research has shown depression and anxiety are the most BC complications [44]. Fear of recurrence, death, and the side effects of treatments disturb the mental state and cause anxiety, negatively affecting the Qol of BC survivors. Our finding demonstrated that although the decreased cortisol levels after intervention were not significant intergroup, the anxiety level was significantly reduced in both yoga groups. There also were significant associations between anxiety level and survival-related genes. Changing the blood indicators of the psychological state, cortisol, may take more than three months. However, yoga is a practical approach for individuals with elevated anxiety levels [45], particularly for BC patients [46]. Although the precise mechanisms of yoga on anxiety are unclear, however, several hypotheses have been proposed. Given that the yoga training was held in groups and perhaps the feeling of empathy, sharing problems, and improving social behaviors could have to some extent, resulted in reducing the anxiety [46]. In addition, yoga exercises include meditation, which leads to improved emotional health through learning how to relax, thereby reduces anxiety. Moreover, from a molecular point, yoga promotes brain health by increasing brain-derived neurotrophic factor (BDNF) [47]) which is a crucial neurotrophin in the management of mood disorders [48]. Therefore, yoga is generally effective in improving mood state, but the manifestation of this condition in the blood takes more time.

Surgery and chemotherapy can exaggerate fatigue, reduce aerobic fitness, and decline the flexibility of the joints, especially the shoulder girdle on the affected side [49]. Collectively, these changes result in a decline in quality of life. Our findings showed significant improvements in flexibility and aerobic fitness following the yoga training program. Consistent with our findings, previous studies reported that yoga training significantly improves shoulder range of motion [50, 51]. Improving the flexibility of the shoulder girdle in the present study is expected due to the nature of yoga movements are stretching-muscular endurance movements. However, improved aerobic fitness with yoga was an interesting finding of this study, given that the intensity of training remained low to moderate during the interventions, and yoga is not primarily specific to improving aerobic fitness. However, the patients' baseline aerobic fitness was low, which could be attributed to fatigue resulting from cancer and chemotherapy. Participating in yoga training for 12 weeks may have sufficient to improve aerobic fitness.

Apoptosis is a sophisticated process that is tightly regulated by the equilibrium of apoptotic and anti-apoptotic genes. NF-κB and p53 are the primary regulators of cell survival and can be activated by the different stimuli; the interactions of these two genes and their downstream pathways are critical for cell fate decisions. Previous research has revealed that physical training and VD supplementation can modify this cellular interaction [19, 31]. Our finding showed that yoga training combined with the high dose of VD supplementation significantly upregulated leukocytes P53 and Bcl2 while no significant downregulation in NF-κB and Bax was found. Although downregulation in NF-κB and Bax were not statistically significant, which might be due to the short duration of the study, it was clinically valuable. Studies reported that treatment with VD down-regulates NF-κB expression in human lymphocytes [20, 21]. Also, Gupta et al. [19] demonstrated that treatment with VD improved cell survival after ultraviolet radiation by increasing p53 expression. This effect was dose-dependent. The up-regulation of the p53 gene induces cell cycle arrest and repair of DNA, hence increased cell survival [52]. Moreover, a study showed a high dose of VD led to an increase in Bcl-2 expression [53]. In this regard, Wang et al. reported incubating VD to thyrocytes induced the expression of Bcl2 but did not affect the expression of Bax [54]. They suggested that an elevated Bcl2/Bax ratio may protect leukocytes from apoptosis. On the other hand, physical exercise may be effective in the apoptosis process. In this regard, Ram et al. [55] compare the lymphocyte apoptotic index and DNA damage of trained yoga practitioners with BC patients. They reported that apoptosis and DNA damage of lymphocytes were less in the yoga group than in the non-yoga group [55]. Therefore, performing yoga with a high dose of VD supplementation, not a low dose, will help improve the function of immune cells by modifying gene expression involved in the apoptosis process.

This study contains some limitations. First, it was difficult to monitor the participants' dietary intake, and we could not quantify the amount of VD taken from the diet. Second, monitoring the intensity of yoga and individualizing yoga's intensity is complicated; however, the Borg scale, which demonstrates the general feeling and the degree of the mental and physical difficulty of the work done, was used, which merits some limitations. We did not observe significant changes in some molecular markers; therefore, further research focusing on longer training intervention time-varying in intensities and higher dosage of VD supplementation is warranted.

## Conclusion and implication

In conclusion, combining yoga training with a high dose of VD supplementation has further benefits on some crucial molecular markers of immune cell survival, physical and psychological status of BC survivors. Therefore, BC survivors would be encouraged to perform yoga training and also simultaneously consume a high dose of VD supplementation, not low doses, to obtain more health benefits.

## Data Availability

Data would be available from the corresponding author on reasonable request.

## Abbreviations

BC: Breast cancer
Qol: Quality of life
IL-6: Interleukin-6
IL-10: Interleukin-10
TNF-α: Tumor necrosis factor-α
PBMCs: Peripheral blood mononuclear cells
VD: Vitamin D
HVD: High dose of VD
Y-HVD: Yoga with a high dose of VD
Y-LVD: Yoga with a low dose of VD
BF%: Body fat percentage
RPE: Rating of perceived exertion
ANCOVA: Analysis of covariance
ES: Effect sizes
